# Full retraction of Kumar DM, Simpkins JW, Agarwal N. Estrogens and neuroprotection in retinal diseases. Mol Vis. 2008;14:1480-6. Review.

**Published:** 2008-11-27

**Authors:** The Editors

**Affiliations:** Emory University, Atlanta, GA

## Introduction

In his capacity as Research Integrity Officer and Vice President for Research of the University of North Texas Health Science Center, Dr. Glenn H. Dillon, Ph.D. requested the full retraction of the above review article (Mol Vis 2008; 14:).

Dr. Dillon states that one of the faculty of University of North Texas Health Science Center, Dr. Lazlo Prokai, found that data from Dr. Prokai's laboratory was included in this review article without his knowledge or attribution. Dr. Dillon states that evidently this was done inadvertently, as Drs. Prokai and Agarwal were collaborators, and that the particular data in question were apparently generated by another individual.

Dr. Agarwal, corresponding author, also requests that this article be retracted, stating the error of inclusion of these data.

On this basis, The Editors formally retract this article from Molecular Vision.

Sincerely,

The Editors

